# Can health insurance protect against out-of-pocket and catastrophic expenditures and also support poverty reduction? Evidence from Ghana’s National Health Insurance Scheme

**DOI:** 10.1186/s12939-016-0401-1

**Published:** 2016-07-22

**Authors:** Genevieve Cecilia Aryeetey, Judith Westeneng, Ernst Spaan, Caroline Jehu-Appiah, Irene Akua Agyepong, Rob Baltussen

**Affiliations:** School of Public Health, College of Health Sciences, University of Ghana, P. O. Box LG 13, Legon, Ghana; Rutgers, Arthur van Schendelstraat 696, Utrecht, Netherlands; Department for Health Evidence, Radboud University Medical Canter, P.O. Box 9101, 6500 HB Nijmegen, Netherlands; African Development Bank, Human Development Department, Tunis, Tunisia

**Keywords:** Health insurance, Catastrophic expenditure, Out-of-pocket expenditure, Poverty reduction, Ghana

## Abstract

**Background:**

Ghana since 2004, begun implementation of a National Health Insurance Scheme (NHIS) to minimize financial barriers to health care at point of use of service. Usually health insurance is expected to offer financial protection to households. This study aims to analyze the effect health insurance on household out-of-pocket expenditure (OOPE), catastrophic expenditure (CE) and poverty.

**Methods:**

We conducted two repeated household surveys in two regions of Ghana in 2009 and 2011. We first analyzed the effect of OOPE on poverty by estimating poverty headcount before and after OOPE were incurred. We also employed probit models and use of instrumental variables to analyze the effect of health insurance on OOPE, CE and poverty.

**Results:**

Our findings showed that between 7–18 % of insured households incurred CE as a result of OOPE whereas this was between 29–36 % for uninsured households. In addition, between 3–5 % of both insured and uninsured households fell into poverty due to OOPE. Our regression analyses revealed that health insurance enrolment reduced OOPE by 86 % and protected households against CE and poverty by 3.0 % and 7.5 % respectively.

**Conclusion:**

This study provides evidence that high OOPE leads to CE and poverty in Ghana but enrolment into the NHIS reduces OOPE, provides financial protection against CE and reduces poverty. These findings support the pro-poor policy objective of Ghana’s National Health Insurance Scheme and holds relevance to other low and middle income countries implementing or aiming to implement insurance schemes.

## Background

The reliance on out-of-pocket expenditure (OOPE) to finance health care is a common feature in many low and middle income countries (LMICs). Households without adequate financial protection face the risk of incurring large unforeseen medical expenditures should they fall ill. These unforeseen expenditures sometimes may lead to indebtedness and reduction in living standards leading to poverty [1–6]. The quest for financial protection to minimize the extent to which households incur catastrophic expenditure (CE) and are pushed into poverty due to high medical spending has received a lot of attention. More recently, health insurance has been put forward as an instrument to provide financial protection and to achieve universal coverage [7–11].

Again, although evidence in many LMICs suggest that the insured sometimes incur high out-of- pocket expenditures, sometimes large enough to push them into catastrophe [9, 12–15], studies on the effect of health insurance on poverty reduction in particular is limited in many LMIC settings, including Ghana. We set out to answer the following questions: (1) Does OOPE for health care lead to poverty at the household level? (2) Does health insurance reduce OOPE, CE and poverty at the household level? This study adds to existing literature on evidence of the effect of health insurance on poverty, OOPE and CE using various econometric techniques.

### Ghana’s National Health Insurance Scheme

In 2004, Ghana began implementation of a National Health Insurance Scheme (NHIS) as part of the government’s policy to minimize the financial burden of OOPE at point of service and to ensure equitable access to health care, particularly among the poor. The NHIS is publically financed by a single pooled National Health Insurance Fund. The fund has three main sources: tax revenue in the form of a 2.5 % VAT which contributes about 70 % of the fund, 2.5 % of contributions of Social Security and National Insurance Trust (SSNIT) contributors who are formal sector workers; and which contributes about 20 % of the fund; and out-of-pocket income adjusted premiums ranging from GHS7.2-GHS48 (US$ 2-US$12) for non SSNIT contributors. In addition, apart from certain exempt groups, a registration fee payment is a requirement before obtaining a new card or renewing an old one. The fund is used to pay for health services, administration of the NHIS and premium exemptions for pregnant women and their newborn, children under 18 years and the aged over 70 years as well as those successfully identified as too poor to pay (i.e. indigent). The challenge remains regarding how to identify the indigents for premium exemptions prior to enrolment [16, 17]. Apart from beneficiaries of the livelihood empowerment against poverty (LEAP) [18], a conditional cash transfer program, the identification of indigents leaves a large amount of discretion to the staff of the district scheme offices.

The scheme requires by law for all nationals resident in the country to enroll. However it has not been possible to enforce compulsory enrolment/re-enrolment given the large informal sector. Also, the annual enrolment and renewal arrangements that require subscribers to present at an enrolment outlet to pay registration fees and/or premiums and then receive an insurance card or have their card endorsed present a barrier to the compulsory enrolment/re-enrolment policy [19].

The purchaser arrangements of the scheme are managed at the national level by the National Health Insurance Authority (NHIA) and peripherally by its regional and district branch offices. Services are provided through contractual arrangements with public and private providers, pharmacies and diagnostic services. The NHIS benefit package covers range of services including outpatient and inpatient care, some aspects of oral health, eye care, maternity care and emergencies. It excludes cosmetic services, HIV anti-retroviral medicines, orthopedics, and organ transplant among others. According to the scheme, over 95 % of disease conditions are covered under the scheme [20].

Since the introduction of the scheme, enrolment has increased from 6 % in 2005 to 38 % in 2014. Similarly, utilization of health services by the insured have also increased. According to Ghana Health Service annual reports, the proportion of OPD attendance by insured clients increased from 55.81 % in 2010 to 82.11 % in 2011, OPD per capita increased from 0.98 in 2010 to 1.14 in 2012. The improvement observed may be largely due to increase in access to health care and increasing demand for health services and existing enrolment on the National Health Insurance Scheme [21, 22].

Similarly, Ghana’s recent living standards survey in 2012 (GLSS VI) revealed that the country’s poverty profile has declined since 2006 from 31.9 % to 24.2 % in 2013, with the Northern, Upper East and Upper West regions recording higher poverty incidence above 70 %. The other regions on average showed a general decline from 24.7 % in 2005 to 20.5 % in 2012 [23].

## Methods

### Study setting, sampling and data collection

This study was part of a bigger research project exploring options to better reach the poor with health insurance. It involved qualitative and quantitative inquiry into identification of barriers to enrolment and implementation of an intervention to improve enrolment. An impact evaluation of the intervention was undertaken, the results of which have been reported elsewhere [24]. Two rounds of survey were conducted in the Eastern (ER) and Central (CR) regions of Ghana in 2009 and 2011 at baseline and endline respectively. These are two adjacent regions in Southern Ghana and are characterized by a mixed rural and urban populations, relatively similar socioeconomic, demographic and cultural characteristics. The central region lies within the coastal belt of the country with population of around 1.8 million and poverty incidence of 23.4 % in 2006. The Eastern region lies to the east of the country with an estimated population of 2.3 million and poverty incidence of 17.8 % in 2006 [25].

With respect to sampling, we drew our sample using a three-stage sampling procedure. The target was to collect information from 3,000 households in total from both regions. First, all the 30 districts from the two regions were selected (i.e. 17 from ER and 13 from CR). Second, one census enumeration area (EA), demarcated by the Ghana Statistical services was randomly selected from each district, using a set of computer generated random numbers [26]. One EA per district was selected in order to measure variations across the districts. Each district is made up of a number of EAs sometimes classified according to communities. Thus an EA can be a whole community and also a community may have more than one EA depending on the size. Third, we mapped and numbered all residential structures within the selected EAs out of which 110 households were randomly selected. A total of 3,300 (110 by 30 EAs) households were involved in our survey with 13,857 individuals at baseline in 2009. In the 2011 follow-up survey 149 households were lost reducing our sample to 3,152 households with 12,810 individuals. Lost households were mainly those that had relocated to other communities or single household members who have died.

We collected data one-time from households in the two periods (i.e. 2009 and 2011) with 1 month recall for information on household expenditure, expenditure on health and income variables. Other variables to which data was collected include insurance status, asset ownership, education, employment, locality, health status, health services patronized, i.e. outpatient and in-patient services among others. We used expenditure rather than income because it has been observed over time that in many developing countries setting, the use of income for socioeconomic measures can be unreliable.

### Analysis

#### Effect of OOPE on CE and poverty

Following the methodology developed by O’Donnell et al. 2008 on the poverty impact of OOPE [2, 27], we analyzed the effect of OOPE on poverty by estimating and comparing the poverty headcount before (pre) and after (post) OOPE for health care were incurred. Following international literature on poverty measurements [28], we defined our poverty line as the estimated consumption level needed to satisfy minimum subsistence need. We set this line at GH¢60.72 (US$ 43.4) which was the mean monthly food expenditure of our sample. Households whose total expenditure was equal to or below this minimum were considered poor. We illustrate the effect of OOPE on poverty using Pen’s parade [27, 29]. The Pen’s parade is a line chart showing the distribution of pre and post OOPE and the poverty line by ranking households in ascending order of total income or expenditure. The parade visualizes the distribution of households that fall below the poverty line due to OOPE.

#### Effect of insurance on OOPE, CE and poverty

All analyses were conducted at the household level applying panel data techniques. We defined OOPE as household health expenditures incurred for both in and out-patient services including expenditure on medicines, transport, diagnostics and under-the- table payments if incurred. We used the methodology proposed by WHO to estimate CE [30, 31] and we defined household spending to be catastrophic if OOPE equaled or exceeded a certain payment capacity threshold i.e. 40 % of non-food consumption expenditure (also known as capacity to pay). Non-food consumption expenditure was calculated as total expenditures less food expenditure. We used the same definition of poverty described in the previous section on effect of OOPE on poverty. The recall period for expenditure data was within the last 30 days prior to the surveys. In all analyses of expenditure, we adjusted for household size using adult equivalence scale formula equivalence size = (household size)^β^ where β = 0.56 [32].

Health insurance has been identified in the literature to be a potentially endogenous variable that determines OOPE, CE or poverty [13, 33–35]. That is, households with relatively high health expenditures are likely to enroll. In addition, poverty is a likely determinant of health insurance enrolment for financial protection. Bearing this in mind, we undertook several types of analyses to test for the potential endogeneity of health insurance in our data and if it exist to address it. First assuming insurance to be exogenous, we applied the random effects model to analyze the effect of health insurance on OOPE and the probit model for the effect of health insurance on CE and poverty with CE and poverty as binary outcome variables. To address the potential endogeneity of health insurance, we used instrumental variable (IV) analysis and applied the two stage least squares (2SLS) for effect of insurance on OOPE and two stage residual inclusion (2SRI) for analysis of effect of insurance on CE and poverty. The 2SRI is the method recommended when the outcome and predictor variables are binary in an IV model. In the 2SLS model, the first stage proceeds by running a linear model of the endogenous variable with identified instruments. In the second stage, the residuals from the first stage are included in a linear model of the outcome variable and other explanatory variables. Similarly in the 2SRI model, the first stage proceeds with running a non-linear regression of insurance on the covariate vector and the instruments. Then the residuals from the first stage regression are incorporated into a non-linear regression of outcome (CE and poverty) on the covariate vector, insurance and the residuals [36–38].

OOPE, CE and poverty can be written as a function of health insurance and other exogenous explanatory variables as follows:

OOPE 2SLS IV model

1 $$ {Y}_{ij}={\beta}_1{I}_{ij}+{\beta}_2{T}_{ij}+{\varepsilon}_{ij} $$

2 $$ {I}_{ij}={\alpha}_1{Z}_{ij}+{\alpha}_2{X}_{ij}+{\mu}_{ij} $$

CE and poverty 2SRI model:

3 $$ \begin{array}{l}C{P}_{ij}^{*}={\beta}_1{I}_{ij}+{\beta}_2{T}_{ij}+{\varepsilon}_{ij}\\ {}C{P}_{ij}=1\kern0.36em  if\;C{P}_{ij}^{*}>0\\ {}C{P}_{IJ}=0\kern0.48em  otherwise\end{array} $$

4 $$ \begin{array}{l}{I}_{ij}^{*}={\alpha}_1{Z}_{ij}+{\alpha}_2{X}_{ij}+{\mu}_{ij}\\ {}{I}_{ij}=1\kern0.36em  if\kern0.48em {I}_{IJ}^{*}>0\\ {}{I}_{ij}=0\kern0.48em  otherwise\end{array} $$

Where Y is out-of-pocket expenditure (OOPE), I is household health insurance status, T is a vector of other independent variables (or covariates). Z is a vector of instrumental variables predicting health insurance but not OOPE. X is a vector of exogenous variables from the OOPE equation. CP is a dummy dependent variable for catastrophic expenditures or poverty. α and β are set of parameters characterizing the function while *i* is the panel variable and *j* the time variable. Following from existing literature, the following control variables were employed in our analysis: Year (dummy for year 2009), locality (dummy for rural locality), out-patient and in-patient use of health service (dummy), consumption expenditure (continuous), household head characteristics including age (continuous), sex, education (four dummies with base of no education) employment status (four dummies with base of casual work) and health status (dummy with base of poor health status).

#### Test for the endogeneity of health insurance and instrumental variables

The Hausman endogeneity test evaluates whether there are systematic differences between the estimates obtained from the naïve (exogenous) model and the IV estimates [39]. Rejecting the null hypothesis suggests the presence of endogeneity and therefore the use of an IV approach. Also, for the IV estimates to improve over the naïve (exogenous) equation estimates, it is fundamental that instruments satisfy two condition: (a) relevance, that is, instruments must be correlated with and have a sufficient explanatory power for the endogenous variable: (b) validity, that is, instruments must not have a direct effect on the outcome in the second stage so that they can be excluded from the main (second stage) equation [33, 40–42].

We identified possible instrumental variables based on theory and existing literature. Our study sample was drawn from a randomized controlled trial to analyze the effect of a community intervention on enrolment, described in the methods section, and we employed household participation in the intervention as an instrumental variable. Because of the randomization process, we considered participation in the intervention to be exogenous of OOPE, CE and poverty. We represented the intervention by a dummy variable if the household belonged to the intervention community. In addition we considered other instruments such as price of insurance, community enrolment rate, location of scheme office, attitude of health personnel towards insured and uninsured client and community beliefs about insurance.

#### Test for random or fixed effects 2SLS

The precondition for using random effects are (i) that observations are drawn randomly from a given population and (ii) that the unobserved effect be distributed independently across X_j_ variables. We used the Durbin-Wu-Hausman test to determine whether to use the random or fixed effects 2SLS analysis. Under this test, the null hypothesis is that the preferred model is the random effects and alternative hypothesis is fixed effects. Rejecting the null hypothesis means fixed effects model is preferred [43].

## Results

### Characteristics of sample

The socio-economic characteristics of households in 2009 and 2011 are presented in Table 1. Since we conducted a repeated survey over the same households, moderate differences were reported in variables such as age, education, sex and occupation. Household nominal welfare levels improved considerably between the survey periods illustrated by an increase in mean monthly incomes from GH¢187 (US$133) in 2009 to GH¢307 (US$219) in 2011. Poverty levels declined from 34 % to 20 % while household enrolment into the NHIS increased from 31 % to 37 %.

**Table 1:** Household socio-economic characteristics

	2009	2011	Difference (*p*-value)
Mean household size	4.211	4.066	0.145 (0.513)
Locality (%)			
Rural	1,876 (60.0)	1,876 (60.0)	0 (1.000)
Urban	1,252 (40.0)	1,252 (40.0)	0 (1.000)
Household head characteristics			
Sex (%)			
Male	2,016 (64.5)	1,970 (63.0)	46 (0.361)
Female	1,112 (35.6)	1,158 (37.0)	46 (0.361)
Marital status (%)			
Never married	508 (16.2)	486 (15.5)	22 (0.496)
Married	1,997 (63.8)	1,975 (63.1)	2 (0.633)
Divorced	623 (19.2)	667 (21.3)	44 (0.256)
Religion			
Christian	2,715 (86.9)	2,776 (88.8)	61 (0.484)
Muslim	195 (6.2)	201 (6.4)	6 (0.948)
Traditional	57 (1.8)	41 (1.3)	16 (0.507)
None	159 (5.1)	107 (3.4)	52 (0.171)
Education (%)			
None	816 (26.1)	816 (26.1)	0 (1.000)
Primary	684 (21.9)	649 (20.8)	35 (0.740)
Secondary	1,341 (42.9)	1,391 (44.5)	50 (0.750)
Tertiary	287 (9.2)	272 (8.7)	15 (0.912)
Occupation (%)			
Farmer/fisherman	1,157 (37.0)	1,200 (38.4)	43 (0.883)
Casual worker	179 (5.7)	228 (7.3)	49 (0.541)
Student/retired	159 (5.1)	176 (5.6)	17 (0.656)
Self/government employed	1,514 (48.5)	1,422 (45.5)	92 (0.699)
Unemployed	116 (3.7)	100 (3.2)	16 (0.584)
Household welfare GH¢ (SE)			
Mean monthly income	187.2 (4.1)	306.8 (5.8)	245.6 (0.000)
Mean monthly expenditure	181.9 (5.6)	358.3 (6.2)	268.1 (0.000)
Headcount poverty	34.4	20.2	27.4 (0.000)
Household health expenditure GH¢ (SE)			
Mean OOPE: OPD	23.0 (1.9)	32.6 (3.4)	25.7 (0.009)
Mean OOPE: IPD	51.0 (6.7)	62.2 (14.4)	53.5 (0.425)
Incidence of catastrophic health expenditure	27 %	12 %	15 (0.000)
Insurance Status (%)			
Insured	986 (31.2)	1,176 (37.5)	190 (0.000)
Uninsured	2,142 (68.5)	1,952 (62.4)	190 (0.000)

Exchange rate GH¢1.45: US$1 SE: Standard errors

### Effect of OOPE on CE and poverty

**Fig. 1:**
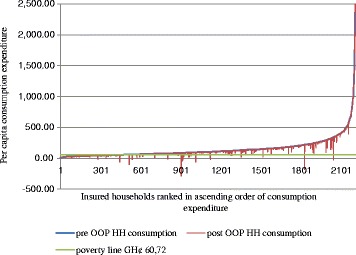
Parade of insured households who fell into poverty due to OOPE

**Fig. 2:**
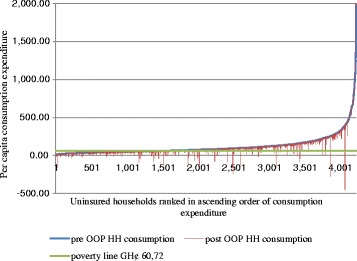
Parade of uninsured households who fell into poverty due to OOPE

Mean OOPE for out-patient services for the entire sample increased from GH¢ 23 to GH¢33 and from GH¢ 51 to GH¢62 for in-patient services between 2009 and 2011. Household incidence of CE declined from 27 % in 2009 to 12 % in 2011 (Table 1). This decline in CE was significantly more for insured than uninsured households. Between 18 % and 7 % of insured households incurred CE and between 36 % and 29 % of uninsured households incurred CE in 2009 and 2011 respectively (Table 2). The effect of poverty on OOPE revealed that some 3–5 % of both insured and uninsured households fell into poverty between 2009 and 2011. Figures 1 and 2 provide an illustration of the effect by insured and uninsured households using the Pen’s parade.

**Table 2:** Poverty effect of OOPE and household health expenditures by insured and uninsured

	2009			2011		
	Insured	Uninsured	|t| > 0	Insured	Uninsured	|t| > 0
Pre-payment headcount poverty (%)	27.9	37.2	0.000	13.8	23.9	0.000
Post-payment headcount poverty (%)	30.8	42.2	0.000	17.1	26.7	0.000
Diff. in pre and post headcount poverty (%)	2.9	4.9	0.006	2.8	3.3	0.421
Catastrophic expenditure (%)	18.4	36.1	0.000	7.1	28.7	0.000
Mean OOPE_OPD (SE)	19.8 (3.1)	27.2 (2.4)	0,058	26.1 (9.2)	53.5 (3.2)	0,001
Mean OOPE_IPD (SE)	42.5 (10.9)	58.5 (8.2)	0.235	65.9 (18.5)	50.2 (11.9)	0.646

*OOPE* Out of pocket expenditure, *OPD* Out-patient services, *IPD* In-patient services

### Effect of insurance on OOPE, CE and poverty

The result from the Durbin-Wu-Hausman test suggests the use of the random effects to analyze OOPE. The Hausman test of endogeneity suggests the presence of endogeneity of insurance and we thus used several instruments to address the issue. We settled on intervention as the main instrumental variable for our analysis based on its best performance on relevance and validity. A summary of the test carried out are provided in Appendix D.

Table 3 provides a summary of the effect of health insurance on OOPE, CE, expenditure and poverty. The full model is provided in the Appendix including the test on validity of instrumental variables used the analyses. Our results revealed that enrolment into health insurance reduces household OOPE by 86 % (=e^-1.984^-1) [44]. The effect of health insurance is protective i.e. insured households were 3 % less likely to incur CE and 7.5 % less likely to fall into poverty.

**Table 3:** Summary results of effect of health insurance on OOPE, expenditure, CE and poverty

	OOPE		CE		POVERTY	
	Coefficient	Probability^†^	Coefficient	*ME*	Coefficient	*ME*
IV estimates						
2-Stage Least Squares	−1.984**	−0.862				
2-Stage Residual Inclusion			−0.637***	−0.031	−0.225***	−0.075

***p < 0.001, **p < 0.01: ME-marginal effects; †calculated as (e^β^-1): effect of dummy variable in a semi-logarithmic equations where β is the regression coefficient

With regard to the other explanatory variables, our findings showed that households who used both in-patient and out-patient services were more likely to incur OOPE. The effect was greatest for households using in-patient services. Also households with good perceived health status were likely to spend significantly less (44 %) on health payments compared to those with poor health status. For CE, households were 5 % more likely to incur CE when using out-patient services while this was 19 % when using in-patient services. Similarly for poverty, use of out-patient services increased the probability of households falling into poverty by 4 % as against 16 % for use of in-patient services. In addition large households and households with unemployed individuals were more likely to fall into poverty and having higher education reduced the incidence of poverty. The details of the results for all models are provided in the Appendix.

## Discussion

Our study was motivated by the policy focus of reaching Ghana’s population with health insurance especially among poor households and the benefits thereof such as reduction in OOPE, CE and poverty over time. We discuss noteworthy findings.

First, enrolment into health insurance reduced household OOPE by 86 %. At the same time, some insured households incurred OOPE sometimes high enough to be catastrophic. In our study, in 2011 the average OOPE for out-patient services were GHS 26.1 and GHS 53.5 for insured and uninsured households respectively and the average OOPE for in-patient services were GHS 65.9 and GHS 60.2 for insured and uninsured households. Thus despite the good news about the protective effect of the NHIS and given that the NHIS policy in theory does not include co-payments, sometimes significant OOPE are taking place especially for inpatient care. One potential explanation is that Ghana’s NHIS benefit package is fairly comprehensive and covers 80–90 % of the most common conditions in Ghana. There are still excluded conditions and these account for the co-payments. However, since our data shows that co-payments are occurring for both in-patient and out-patient services of all kinds including those covered by insurance, clearly the conditions excluded are not the only reason for out-of-pocket spending and may not even be the major reason. Though in theory there are not supposed to be co-pays or balance billing under the NHIS, anecdotal reports and some studies have reports of insured clients being given prescriptions to purchase medicines in the benefit package, with the reason that these are not available in stock and need to be paid for privately with related complaints by providers that NHIS indebtedness makes them unable to stock medicines [45]. There is similarly anecdotal evidence of insured clients preferring to leave their cards at home and rather pay out of pocket to get faster treatment. There are similar reports of informal fees. Any or a mixture of these reasons could explain the high out of pocket expenditures. A similar study in Uganda on why CE remained the same for the poor after removal of user fees found that informal payments in public facilities increased to ‘compensate’ providers for revenue lost from user fees [46, 47].

Second, one of the key objectives of health insurance, as stated in the introduction, is to provide financial protection and minimize the extent to which households incur CE due to health spending. From our analysis, we found that health insurance was protective against households incurring CE. We demonstrated that insured households were 3 % less likely to fall into CE. Though the marginal effects of insurance is relatively low, our outcome is a promising one as it shows the potential of Ghana’s NHIS in achieving financial protection for its enrolled clients. The international evidence on the financial protection effect of insurance on CE is mixed. For example Galarraga et al. 2010 [13] found that households in Mexico were 54 % less likely to incur CE, Saskena et al. 2006 [12] reported a decline in CE from 19.1 % to 8.5 % for households in the lowest quintile in Kenya, Nguyen et al. 2011 [15] also reported a reduction in CE in Ghana. Studies by Dong et al. 1999 and Wagstaff et al. 2008 [48, 49] however found insurance to lead to increased out-of-pocket and catastrophic payments in China because insurance encouraged people to seek care when sick and also to seek care from higher-level providers.

Third, despite a general decline in poverty between 2009 and 2011 (i.e. 34 % to 20 %), OOPE on health care pushed some 3–5 % of households into poverty supporting existing evidence. Expenditures related to in-patient services were the main drivers of households further into poverty [2, 50, 51]. Analyzing the effect of insurance in providing financial protection against poverty, our study revealed that being insured reduces household’s probability of falling into poverty by 7.5 %. This outcome is of relevance to policy as it shows the potential of health insurance to contribute to poverty reduction. Though there is limited evidence of the effect of health insurance on poverty, our findings compares to some studies where impact of health insurance on poverty were discussed through the channel of reduced CE leading to reduction in poverty. They included studies in Senegal [52] and Vietnam [11].

Our study has a number of limitations in interpretation of the results. First, our short recall period of 30 days prior to the survey may underestimate health payments if utilization during that period was low. Nonetheless, shorter recall period enabled respondents to provide reliable payment responses. Second, income was not used as a measure of people’s wealth in this study for the following reasons. A majority of households visited were informal sector workers with unstable income. The instability of this variable may inform biased socioeconomic status for the household at the point of data collection. Socially and culturally, people have reservations for reporting their incomes for fear of taxes and other beliefs. People are also likely to under report or over report depending on the impression they wish to make on the interviewer. Some studies have applied subjective income as a measure of household wellbeing to address this limitation [53, 54]. Third our model on OOPE was a random effects model and it may be possible that issues of unobserved heterogeneity may be present and may have affected our findings. Yet statistical tests indicated the use of random effects model for analysis of OOPE. This may have accounted for the large effect of insurance on OOPE [43]. For the analysis of the effect of insurance on CE and poverty (binary outcomes), to the best of our knowledge only random effects and population average models are available for longitudinal probit model estimation and were employed as such.

## Conclusions

In conclusion, our study provides further confirmation of the existing body of evidence that high OOPE pushes people into poverty. Importantly, it also shows that the Ghana NHIS is effectively providing significant financial protection for households and reducing their likelihood of incurring CE and falling into poverty. The NHIS is therefore achieving to some extent its pro-poor objectives. However, insured clients are still making out-of-pocket payments even though the level is significantly lower than the uninsured clients. In some cases the OOPE payments of insured clients are large enough relative to their income to be catastrophic and tip households into poverty. Hence there is room for improving the extent to which the NHIS is attaining its pro-poor objectives. Further work is required to understand the issues, design and implement appropriate interventions. Indeed, currently, the NHIS in Ghana is has employed some researchers, including a co-author of this manuscript to gather evidence on providers and insurers engagements to confirm or dispute issues such as the insured having to pay for services and also concerns of under-the-the table payment by the insured. These observations and lessons from this study are of relevance to inform considerations in design and implementation of the NHIS in Ghana and also to other LMICs considering introduction of health insurance to achieve effective financial protection for their citizens.

## Abbreviations

2SLS, two-stage-least squares; 2SR1, two-stage residual inclusion; CE, catastrophic expenditures; IV, instrumental variable; LMIC, Lower Middle Income Country; NHIA, National Health Insurance Authority; NHIS, National Health Insurance Scheme; OOPE, out-of-pocket expenditure.

## Appendix

**Table 4:** Effect of insurance on household out-of-pocket expenditure (OOPE)

Dependent variable: Log OOPE	(1)	(2)
	Naïve model	2SLS
Insurance	−0.749***	−1.984**
	(0.063)	(0.647)
Log income	0.279***	0.355***
	(0.045)	(0.060)
Age	0.001	0.012*
	(0.002)	(0.006)
Male	−0.011	−0.124
	(0.067)	(0.089)
Household size	−0.047***	−0.044**
	(0.012)	(0.013)
Rural locality	−0.238***	−0.204**
	(0.068)	(0.070)
Year 2009	0.323***	0.459***
	(0.069)	(0.104)
Good health status	−0.487***	−0.574***
	(0.097)	(0.121)
Use of out-patient services	0.317***	0.470***
	(0.061)	(0.106)
Use of in-patient services	0.964***	1.009***
	(0.141)	(0.148)
Occupation: Farmer	−0.049	−0.164
	(0.095)	(0.118)
Government/Self-employed	−0.024	−0.066
	(0.096)	(0.109)
Unemployed	−0.049	−0.099
	(0.163)	(0.182)
Education level: Primary	−0.129	−0.155
	(0.085)	(0.088)
Secondary	−0.039	0.100
	(0.079)	(0.108)
Tertiary	0.030	0.418
	(0.124)	(0.248)
Constant	1.270***	1.050**
	(0.289)	(0.333)
Observations	2,318	2,301
R-squared		0.153
Number of households	1,844	1,833

Standard errors in parentheses *** *p* < 0.001, ***p* < 0.01, *p < 0.05; 2SLS-2 Stage Least Squares

**Table 5:** Effect of insurance on catastrophic expenditure

	Naive model		2SRI	
	Coefficient	ME	Coefficient	ME
Insurance	−0.739***	−0.036	−0.637***	−0.031
	(0.098)		(0.101)	
Age	0.001	0.000	0.008**	0.000
	(0.002)		(0.003)	
Male	−0.106	−0.006	−0.161*	−0.009
	(0.069)		(0.071)	
Household size	−0.039**	−0.002	−0.037*	−0.002
	(0.015)		(0.015)	
Rural locality	−0.086	−0.004	−0.080	−0.004
	(0.070)		(0.071)	
Year 2009	0.632***	0.037	0.581***	0.033
	(0.071)		(0.073)	
Good health status	−0.959***	−0.122	−1.046***	−0.138
	(0.119)		(0.125)	
Use of out-patient services	0.501***	0.046	0.543***	0.050
	(0.149)		(0.149)	
Use of in-patient services	1.235***	0.152	1.410***	0.188
	(0.078)		(0.086)	
Occupation: Farmer	0.043	0.002	−0.037	−0.002
	(0.105)		(0.109)	
Government/Self employed	−0.074	−0.004	−0.085	−0.004
	(0.109)		(0.109)	
Unemployed	−0.114	−0.006	−0.169	−0.008
	(0.176)		(0.176)	
Education: Primary	−0.128	−0.007	−0.132	−0.007
	(0.085)		(0.086)	
Secondary	−0.284***	−0.016	−0.170*	−0.009
	(0.083)		(0.085)	
Tertiary	−0.294*	−0.013	−0.013	−0.001
	(0.148)		(0.162)	
Residual			−1.034***	−0.057
			(0.236)	
Constant	−0.990***		−0.936***	
	(0.205)		(0.211)	
Observations	6,448		6,406	

Standard errors in parentheses ****p* < 0.001, ***p* < 0.01; **p* < 0.05: ME- marginal effects 2SRI-2-Stage Residual Inclusion

**Table 6:** Effect of insurance on poverty

	Naive model		2SRI	
	Coefficient	ME	Coefficient	ME
Insurance	−0.289***	−0.096	−0.225***	−0.075
	(0.040)		(0.043)	
Age	0.001	0.001	0.005***	0.002
	(0.001)		(0.002)	
Male	−0.200***	−0.069	−0.234***	−0.081
	(0.041)		(0.042)	
Household size	0.056***	0.019	0.058***	0.019
	(0.008)		(0.008)	
Rural locality	0.285***	0.101	0.290***	0.102
	(0.041)		(0.041)	
Year 2009	0.476***	0.161	0.436***	0.147
	(0.034)		(0.035)	
Good health status	−0.131	−0.046	−0.179*	−0.063
	(0.078)		(0.079)	
Use of out-patient services	0.019	0.006	0.114*	0.040
	(0.047)		(0.052)	
Use of in-patient services	0.406**	0.151	0.429**	0.160
	(0.133)		(0.134)	
Occupation: Farmer	−0.030	−0.101	−0.088	−0.029
	(0.060)		(0.061)	
Government/Self employed	−0.264***	−0.089	−0.280***	−0.095
	(0.060)		(0.060)	
Unemployed	0.322**	0.117	0.282**	0.102
	(0.106)		(0.108)	
Education: Primary	−0.241***	−0.079	−0.240***	−0.079
	(0.052)		(0.052)	
Secondary	−0.356***	−0.119	−0.291***	−0.098
	(0.046)		(0.049)	
Tertiary	−0.997***	−0.248	−0.811***	−0.216
	(0.091)		(0.100)	
Residual			−0.608***	−0.208
			(0.136)	
Constant	−0.408**		−0.363**	
	(0.126)		(0.127)	
Observations	6,448		6,406	

Standard errors in parentheses ****p* < 0.001, ***p* < 0.01; **p* < 0.05: ME- marginal effects 2SRI-2-Stage Residual Inclusion

**Table 7:** Summary of tests for choice of model, endogeneity of insurance and instrumental variables for OOPE

Test	Explanation	Results	Comment
Durbin-Wu-Hausman test for choice of model	H0: Random effects is preferred	Chi-sq (15) = 14.70 *p*-value = 0.4733	Did not reject null hypothesis hence random effects is preferred.
Endogeneity test: Wu-Hausnam F test Durbin-Wu-Hausman chi sq-test	H0: variable is exogenous. Rejection of H0 means that the variable is endogenous and instruments are needed	12.58 F (1,2283)	Insurance is endogenous. IV estimates appropriate.
		*p*-value = 0.00040	
		12.61 Chi-sq (1)	
		*p*-value 0.00038	
Kleiberg-Paap test for under-identification (Anderson canon. correlation LM statistic)	H0: model is under-identified. Rejection of H0 implies that the model is well identified.	LM statistic 225.565	Instruments well identified.
		Chi-sq (4) *p*-value = 0.000	
Weak instrument test (Cragg-Donald Wald F statistic)	H0: weakly identified. Rejection of H0 & t statistic >10 (=rule of the thumb) implies that the model is not weak.	F statistic 61.976 5 % maximal IV relative bias = 16.85	Instruments not weak.
Sargan-Hansen J statistic	Test if instruments are uncorrelated with error term. Rejection of H0: instruments are invalid. No problem	Statistic = 2.548	Null hypothesis is not rejected implying that the instruments are valid
		Chi-sq = (3)	
		*P*-value = 0.4667	
